# Efficiency and Safety of Endoscopic Injection Sclerotherapy With Ligation for Esophageal Varices: A Retrospective Study.

**DOI:** 10.1002/deo2.70313

**Published:** 2026-05-04

**Authors:** Keita Maki, Hiroaki Haga, Kyoko Hoshikawa, Tomohiro Katsumi, Fumiya Suzuki, Fumi Uchiyama, Yoshiyuki Ueno

**Affiliations:** ^1^ Department of Gastroenterology Yamagata University Faculty of Medicine Yamagata Japan

**Keywords:** adverse events, endoscopic injection sclerotherapy, endoscopic injection sclerotherapy with ligation, esophageal varices, procedure time

## Abstract

**Objectives:**

Endoscopic injection sclerotherapy with ligation (EISL) is a combined endoscopic treatment that involves endoscopic injection sclerotherapy (EIS) and endoscopic variceal ligation. This study aimed to compare the efficacy of conventional EIS and EISL in treating esophageal varices and the usefulness of EISL.

**Methods:**

A total of 104 cases treated with conventional EIS and 44 cases treated with EISL for esophageal varices were included in the study. The procedure duration, intra‐injection volume, and para‐injection volume, the success rate of intravascular injection based on variceal type, recurrence‐free survival, and post‐treatment adverse events were assessed.

**Results:**

The procedure duration was significantly shorter in the EISL group (13.1 ± 7.2 min) than in the conventional EIS group (20.7 ± 8.0 min; *p* < 0.0001). Additionally, the para‐injection volume per session was lower in the EISL group (0.7 ± 1.0 mL) than in the conventional EIS group (1.2 ± 1.1 mL; *p* = 0.0426). The intravascular injection success rate was higher in the EISL group for all variceal morphologies, particularly for F1/F2 esophageal varices. In terms of post‐treatment adverse events, the frequency of fever was lower in the EISL group than in the conventional EIS group (*p* = 0.008). There was no significant difference in recurrence‐free survival between the conventional EIS and EISL groups.

**Conclusions:**

Compared with conventional EIS, EISL required shorter procedure times and reduced paravariceal leakage of the injected sclerosant. EISL also demonstrated higher success rates, particularly for small varices, and fewer post‐treatment adverse events.

## 1 Introduction

Esophageal variceal rupture is a serious complication of liver cirrhosis [1]. Approximately 30% of patients with liver cirrhosis have esophageal varices at the time of diagnosis [2], and the lifetime incidence of esophageal varices in these patients is estimated to be between 80% and 90% [3, 4]. Endoscopic treatment is the primary approach for managing esophageal varices, both in cases of rupture and for elective treatment [5, 6]. In both Japan and the West, endoscopic variceal ligation (EVL) is the first‐line treatment for esophageal variceal rupture [7], effectively controlling active bleeding in approximately 90% of cases [8]. For preventive treatment, Western guidelines (American Association for the Study of Liver Diseases, Baveno VII) recommend a combination of EVL and nonselective β‐blockers [1, 9].

Endoscopic injection sclerotherapy (EIS) is a treatment that involves using a sclerosing agent to block blood flow to esophageal varices, leading to a lower recurrence rate compared to EVL [10, 11, 12]. In Japan, EIS is the first choice for preventive treatment of esophageal varices.

Endoscopic injection sclerotherapy with ligation (EISL) was introduced by Nishikawa et al. in 1994 [13]. The procedure involves performing EIS with the EVL device in place, followed by EVL at the puncture site immediately after injecting the sclerosing agent [14, 15]. According to portal hypertension guidelines, EISL is recommended for pipeline esophageal varices [16], although other guidelines do not clearly define its indications. The reported advantages of EISL include fewer treatment sessions required to achieve eradication of esophageal varices compared with conventional EIS [17] and a shorter endoscopic procedure time [18]; however, the available evidence remains limited. This study aimed to evaluate the clinical efficacy and safety of EISL compared with conventional EIS.

## 2 Methods

### 2.1 Study Design and Patient Inclusion Criteria

All endoscopic sclerotherapy for esophageal varices at Yamagata University Faculty of Medicine has been performed using EISL since April 2023. The study included 104 cases treated with conventional EIS between April 2014 and March 2023 and 44 cases treated with EISL between April 2023 and October 2025. All EIS procedures were performed without a distal attachment, which was defined as conventional EIS in this study.

The study included patients with liver cirrhosis scheduled for prophylactic treatment of esophageal varices with conventional EIS or EISL and those with moderate or large varices (F2 or F3) and/or red color sign (RC) of varices.

Patients with Child‐Pugh Grade C, total bilirubin levels of 4 mg/dL or higher, hepatocellular carcinoma with portal vein invasion (Vp3/Vp4), uncontrolled hepatocellular carcinoma, and renal failure (serum creatinine > 2 mg/dL) were excluded from this study.

Clinical data collected at the time of esophageal varices treatment included age, sex, liver background, modified albumin‐bilirubin grade, location and form of esophageal varices, presence or absence of gastric varices, color of esophageal varices, presence or absence of RC sign, presence or absence of liver cirrhosis, presence or absence of hepatocellular carcinoma, and blood biochemistry.

Regarding the form of esophageal varices, the largest form was recorded if multiple esophageal varices were observed.

### 2.2 Conventional EIS and EISL Procedure

Conventional EIS and EISL were conducted using a GIF‐Q260J endoscope (Olympus Medical Systems, Tokyo, Japan), a 6‐cm balloon for endoscope attachment (Create Medic, Kanagawa, Japan), and a 23‐gauge injection needle (TOP Corporation, Tokyo, Japan). For EISL, an endoscopic esophageal variceal ligation set (SB Kawasumi Laboratories, Kanagawa, Japan) and an overtube (TOP Corporation, Tokyo, Japan) were used.

In conventional EIS, endoscopic procedures were performed without a distal attachment. After identifying the esophageal varix, approximately 25–30 mL of air was injected into the balloon attached to the endoscope to block the blood flow of the esophageal varices.

After puncturing the esophageal varices and confirming backflow of blood, a 5% solution of EOI (equal parts of monoethanolamine oleate [EO: Oldamin; ASKA Pharmaceutical Co., Ltd., Tokyo, Japan] and the non‐ionic iodine‐containing contrast agent iopamidol) was injected under fluoroscopic guidance. The maximum dose of EOI was set at 0.4 mL/kg. If esophageal varices remained suitable for treatment and the maximum amount of EOI was not exceeded, multiple varices were treated in the same session. In the conventional EIS group, the puncture site was compressed with an endoscope‐mounted balloon for several minutes after EOI injection to control bleeding.

**FIGURE 1:**
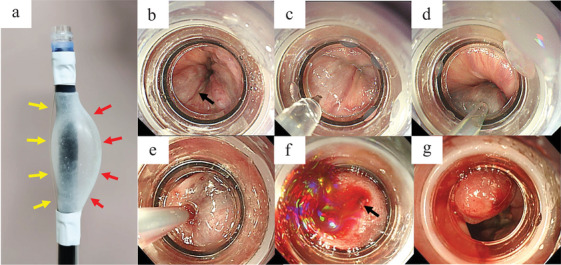
Treatment procedures for endoscopic injection sclerotherapy with ligation (EISL). (a) Fixation of the endoscopic variceal ligation (EVL) device string. The EVL device string (yellow arrow) was fixed on the side opposite to the area of maximal balloon inflation (red arrow). (b) identification of esophageal varices (arrow), (c) fixation of esophageal varices using an EVL device, (d) EOI injection into esophageal varices, (e) Approaching the puncture site with the endoscope, (f) withdrawal of the puncture needle (puncture site, arrow), and (g) EVL at the puncture site.

In the EISL group, an overtube was first placed in the esophagus. After temporary withdrawal of the endoscope, a balloon was attached 1–2 cm proximal to the endoscopic tip, and the EVL device was positioned at the tip of the scope. Regarding placement of the EVL device, after the balloon was inflated, the EVL device string was fixed on the side opposite to the area of maximal balloon inflation (Figure 1a). This modification prevents interference of the EVL device thread within the overtube by reducing slack caused by balloon inflation. After identification of the esophageal varices (Figure 1b), the balloon was inflated. The esophageal varix was fixed by EVL device (Figure 1c), then puncture of the varix was performed, followed by EOI injection (Figure 1d). After EOI injection, suction was applied by advancing the endoscope while carefully preventing needle dislodgement (Figure 1e), and the puncture needle was withdrawn almost simultaneously with the initiation of suction (Figure 1f). Following needle removal, EVL was performed at the puncture site (Figure 1g). After EVL, the balloon was deflated. EVL was limited to hemostasis at the puncture site and was not applied to any other sites.

Endoscopic observation was conducted one week after treatment. If any varices suitable for treatment remained, sclerotherapy was additionally performed. If no suitable varices were found, consolidation therapy (aethoxysklerol) was administered to complete the treatment. All conventional EIS and EISL procedures were carried out by endoscopists and doctors with over 3 years of experience in EIS. Sedation with midazolam and analgesia with pentazocine were used during the procedures.

### 2.3 Definition of Intra‐injection Volume and Para‐injection Volume

The injected EOI volume was classified into intra‐injection volume and para‐injection volume. The intra‐injection volume was defined as the amount of EOI that entered the intravascular space, as confirmed by fluoroscopic visualization of contrast flow into the blood vessels. The para‐injection volume was defined as the amount of EOI that remained in the perivariceal area on fluoroscopy.

### 2.4 Definition of Procedure Duration and Treatment Success Rate (intra‐injection success rate)

Procedure duration was defined as the interval between immediately before needle insertion into the esophageal varices and scope withdrawal. Negative pressure was applied during the injection of the sclerosing agent into the blood vessels, and backflow was confirmed before the injection. The treatment success rate (intra‐injection success rate) was calculated by dividing the number of successful intravenous injections by the total number of esophageal varices punctures. Successful intravenous injection was defined by visualization of the punctured varix; reaching the supplying vessels (e.g., the left gastric vein) under fluoroscopy was not required.

When intravascular injection was initially confirmed, but extravasation occurred later during the same puncture, the injection was classified as a successful intra‐injection. Each puncture was counted independently when the needle was withdrawn, and a repuncture was required, regardless of whether the same esophageal varix was targeted.

The esophageal varices included in the analysis were not limited to the most prominent varix; all esophageal varices that were punctured during the procedures were included. In addition, esophageal varices treated in all treatment sessions were included in the analysis.

### 2.5 Follow‐up and Evaluation of Outcomes

The primary follow‐up outcomes included endoscopy at 3, 6, and 12 months post successful esophageal variceal treatment, with subsequent assessments every 6 months. Successful treatment was indicated by the absence of varices (F0) and RC signs (RC0) on endoscopy. Variceal recurrence was defined as the reappearance of varices necessitating retreatment (F2 or greater varices and/or the presence of RC sign) or recurrent esophageal variceal bleeding. Recurrent bleeding was confirmed by endoscopy and defined as esophagogastric variceal bleeding. Esophageal varices with F1RC0 were analyzed as treatment failure, although they were not regarded as recurrence.

### 2.6 Evaluation of Adverse Events Following Endoscopic Treatment

After conventional EIS or EISL, patients were monitored for adverse events, including physical symptoms (chest heaviness/frontal chest pain, fever, transient dysphagia, hematuria, epigastric pain, nausea, ascites, hiccups, skin rash), abnormal blood test results (transient renal dysfunction, renal dysfunction, encephalopathy), and endoscopic findings (esophageal ulcer, esophageal submucosal hematoma). Fever was defined as an axillary body temperature of ≥38.0°C within 24 h after the procedure.

### 2.7 Statistical Analysis

Recurrence‐free survival was analyzed using Kaplan–Meier curves, and survival curves were compared between the conventional EIS and EISL groups using the log‐rank test. Patients in the conventional EIS and EISL groups were compared using the t‐test or the Mann–Whitney U test for continuous variables and the χ^2^ or Fisher's exact test for categorical variables. Statistical analyses were conducted using GraphPad Prism 9 software, with a significance threshold set at *p* < 0.05.

## 3 Results

### 3.1 Characteristics of Patients With Esophageal Varices (Table 1)

A total of 148 cases of esophageal varices were identified, with 104 cases in the conventional EIS group and 44 cases in the EISL group. The median age of patients in both groups was 67 and 68 years, respectively. The majority of patients in both groups had modified albumin‐bilirubin Grade 2b, and most cases were classified as F2 and RC1.

We performed conventional EIS for esophageal varices until 2023, after which we adopted EISL. The median years of physician experience were 13 years (range, 5–22) for conventional EIS and 8 (range, 6–16) for EISL (*p* < 0.001).

### 3.2 Comparison of Procedure Duration, Intra‐injection Volume, and Para‐injection Volume Between the Conventional EIS and EISL Groups (Figure 2)

**FIGURE 2:**
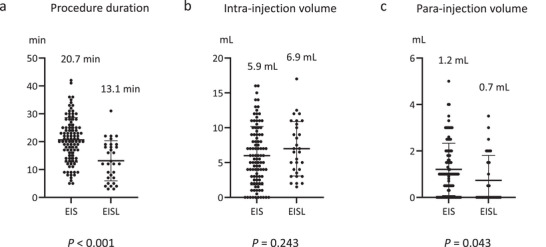
Comparison of procedure duration, intra‐injection volume, and para‐injection volume between the conventional endoscopic injection sclerotherapy (EIS) and endoscopic injection sclerotherapy with ligation groups (EISL). (a) Procedure duration, (b) intra‐injection volume, and (c) para‐injection volume.

The procedure duration was significantly shorter in the EISL group (13.1 ± 7.2 min) than in the conventional EIS group (20.7 ± 8.0 min; *p* < 0.001, Figure 2a ). The intra‐injection volume was 5.9 ± 4.2 mL in the conventional EIS group and 6.9 ± 3.9 mL in the EISL group, with no significant difference observed (*p* = 0.243, Figure 2b). However, the para‐injection volume was 1.2 ± 1.1 mL in the conventional EIS group and 0.7 ± 1.0 mL in the EISL group (*p* = 0.043, Figure 2c), resulting in reduced paravariceal leakage of the injected sclerosant.

### 3.3 Comparison of Successful Puncture Rates Based on Esophageal Varix Morphology Between the Conventional EIS and EISL Groups (Figure 3)

The overall treatment success rate for esophageal varices was 66.4% in the conventional EIS group (354 punctures and 235 intra‐injections) and 83.1% in the EISL group (89 punctures and 74 intra‐injections). The treatment success rates for the conventional EIS group for F3/F2/F1 were 91.2% (57 punctures and 52 intra‐injections), 66.9% (236 punctures and 158 intra‐injections), and 41% (61 punctures and 25 intra‐injections). On the other hand, the treatment success rates for the EISL group were 100% (13 punctures and 13 intra‐injections), 80% (55 punctures and 44 intra‐injections), and 81% (21 punctures and 17 intra‐injections). The treatment success rate was higher in the EISL group than in the conventional EIS group for both forms, with a notable difference in the puncture success rate for F1/F2 esophageal varices.

**FIGURE 3:**
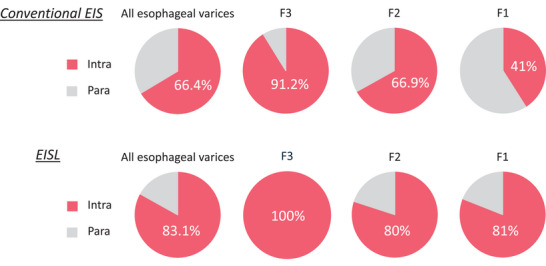
Comparison of successful puncture rates based on esophageal varix morphology between the conventional endoscopic injection sclerotherapy (EIS) and endoscopic injection sclerotherapy with ligation groups (EISL).

### 3.4 Comparison of Recurrence‐free Survival for Esophageal Varices Between the Conventional EIS and EISL Groups (Figure 4)

The median follow‐up period was 840 days (range, 126–2144 days) for the conventional EIS group and 420 days (range, 125–780 days) for the EISL group. No significant difference was found in the 2‐year recurrence‐free survival between the two groups (*p* = 0.802).

**FIGURE 4:**
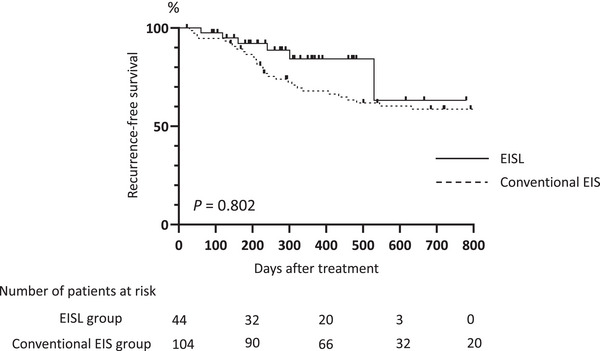
Comparison of recurrence‐free survival for esophageal varices between the conventional endoscopic injection sclerotherapy (EIS) and endoscopic injection sclerotherapy with ligation groups (EISL).

### 3.5 Comparison of Post‐treatment Adverse Events Between the Conventional EIS and EISL Groups (Table 2)

Adverse events following conventional EIS or EISL were assessed. The most frequent symptoms included chest heaviness/frontal chest pain (21.6%), fever (16.9%), and transient dysphagia (7.4%). The incidence of fever was higher in the conventional EIS group at 22.1% compared to 4.5% in the EISL group (*p* = 0.008). Two cases of esophageal submucosal hematoma were observed only in the conventional EIS group (1.9%), both with low platelet counts, showing extensive submucosal hematomas.

## 4 Discussion

This study revealed that compared with conventional EIS, EISL required a shorter treatment duration and led to reduced paravariceal leakage of the injected sclerosant. Additionally, EISL demonstrated a higher treatment success rate than conventional EIS, particularly for small varices such as F1/F2. Furthermore, EISL resulted in fewer post‐treatment adverse events, such as fever, than conventional EIS.

Previous studies have shown that EISL reduces the risk of bleeding from the puncture site by incorporating EVL at the puncture site and extends the retention time of the sclerosing agent in the blood vessel, shortening the procedure duration [19].

This study highlights the benefits of EISL over conventional EIS in terms of the amount of sclerosing agent injected inside and outside esophageal varices, treatment success rates based on variceal morphology, recurrence‐free survival period, and post‐treatment adverse events. The study periods for conventional EIS and EISL differed, and the endoscopists performing the procedures were not identical. Therefore, we compared the duration of endoscopic experience between groups and observed that the endoscopists in the conventional EIS group had significantly longer experience than those in the EISL group (13 [5–22] vs. 8 [6–16] years; *p* < 0.001). Accordingly, the superior outcomes of EISL observed in this study are unlikely to be explained by differences in physician experience.

The EISL group exhibited a shorter endoscopic procedure duration (13.1 ± 7.2 min) than the conventional EIS group (20.7 ± 8.0 min; *p* < 0.001). This difference in procedure duration is attributed to the variances in hemostasis techniques, with EVL being quicker than balloon compression. The volume of para‐injection per session was lower in the EISL group (0.7 ± 1.0 mL) than in the conventional EIS group (1.2 ± 1.1 mL; *p* = 0.043). In the EISL group, the use of the EVL device to contact varices resulted in the suppression of respiratory fluctuations and esophageal peristalsis, improving visualization. This, in turn, reduced needle tip displacement during puncture and minimized leakage outside the blood vessel.

In terms of treatment success rates, the EISL group outperformed the conventional EIS group in all categories. The difference in puncture success rates was particularly notable for F1/F2 esophageal varices. The application of the EVL device to secure varices proved effective in enhancing puncture accuracy and creating tension in the varices, facilitating the puncture of thin varices.

Adverse events following conventional EIS or EISL included a lower incidence of fever in the EISL group (*p* = 0.008). This was attributed to the shorter endoscopic procedure duration with EISL, reducing patient burden. Esophageal submucosal hematoma was only observed in the conventional EIS group. In both cases of submucosal hematoma, which were associated with low platelet counts, EVL at the puncture site may help prevent esophageal submucosal hematoma.

**FIGURE 5:**
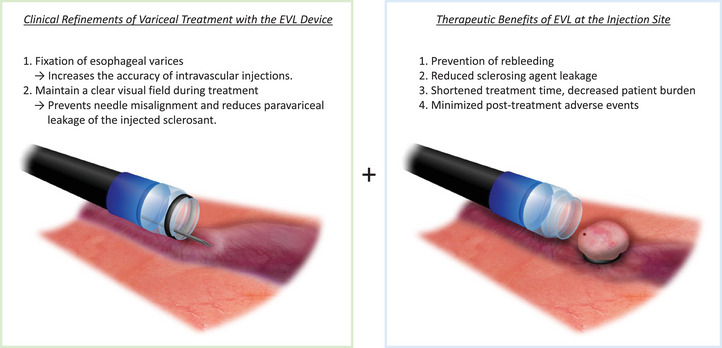
Endoscopic injection sclerotherapy with ligation (EISL) provides two major advantages: a clinical refinement of variceal treatment through the use of an endoscopic variceal ligation (EVL) device, and the therapeutic benefit of EVL applied at the injection site.

EISL offers two major advantages. First, the EVL device fixes the esophageal varices, thereby enhancing the accuracy and success rate of intravascular injections. Moreover, it maintains a clear visual field during treatment, which prevents needle misalignment and reduces paravariceal leakage of the injected sclerosant. Second, EVL at the injection site prevents rebleeding and sclerosant leakage, thereby shortening procedure time and reducing patient burden and post‐treatment adverse events (Figure 5).

While there was no significant difference in the 2‐year recurrence‐free survival between the conventional EIS and EISL groups (*p* = 0.802), variations in treatment duration and background liver disease proportions for conventional EIS and EISL may have influenced these outcomes.

As for limitations, all conventional EIS procedures were performed without using the hood method, which may have influenced treatment success rates compared to facilities that employ this technique. In addition to the presence or absence of a distal attachment, the operator learning curve may have contributed to the improved outcomes observed with EISL. As EISL was introduced later than conventional EIS at our institution, increasing operator experience and technical refinement over time could have influenced the procedural success rates. Therefore, the potential impact of temporal improvements in operator skill cannot be completely excluded.

In conclusion, compared with conventional EIS, EISL required a shorter procedure time and demonstrated reduced paravariceal leakage of the injected sclerosant. Moreover, EISL achieved higher success rates, particularly for small varices (F1/F2), and was associated with fewer post‐treatment adverse events such as fever.

## Author Contributions

*Conceptualization*: Keita Maki and Hiroaki Haga; *methodology*: Keita Maki; *validation*: Keita Maki and Hiroaki Haga; *formal analysis*: Keita Maki; *investigation*: Keita Maki, Hiroaki Haga, Kyoko Hoshikawa, Tomohiro Katsumi, Fumiya Suzuki, Fumi Uchiyama, and Yoshiyuki Ueno; *resources*: Hiroaki Haga, Kyoko Hoshikawa, Tomohiro Katsumi, Fumiya Suzuki, Fumi Uchiyama, and Yoshiyuki Ueno; *data curation*: Keita Maki; *writing – original draft preparation*: Keita Maki; *writing – review and editing*: Keita Maki; *supervision*: Hiroaki Haga; *project administration*: Hiroaki Haga All authors have read and agreed to the published version of the manuscript.

## Funding

This study did not receive any financial support or grant funding.

## Ethics Statement

This retrospective study was approved by the Ethics Review Committee of Yamagata University School of Medicine (approval number: 2019–316).

## Consent

All patients provided informed consent for the use of their clinical information for research purposes.

## Conflicts of Interest

The authors declare no conflicts of interest.

5

**TABLE 1:** Patient characteristics of those who underwent conventional endoscopic injection sclerotherapy (EIS) and endoscopic injection sclerotherapy with ligation (EISL).

Characteristics (*n* = 148)	EIS group (*n* = 104)	EISL group (*n* = 44)	*p*‐value
Age, median (range)	67 (16–85)	68 (19–91)	0.117
Sex, male/female	70/34	25/19	0.262
**Etiology, *n* (%)**			
HBV/HCV/non‐B, non‐C/alcohol/MASH/AIH/PBC/IPH/EPVO/after liver resection	1(1)/18(17.3)/2(1.9)/45(43.3)/15(14.4)/4(3.8)/10(9.6)/2(1.9)/4(3.8)/3(2.9)	2(4.5)/4(9.1)/1(2.3)/14(31.8)/11(25)/2(4.5)/6(13.6)/2(4.5)/1(2.3)/1(2.3)	0.445
**mALBI grade, *n* (%)**			
Grade 1/Grade 2a/Grade 2b/Grade 3	25(24)/25(24)/49(47.1)/5(4.8)	8(18.2)/9(20.4)/23(52.3)/4(9.1)	0.628
Location (Ls/Lm/Li), *n* (%)	31(29.8)/63(60.6)/10(9.6)	11(25)/23(52.3)/10(22.7)	0.127
Form (F1/F2/F3), *n* (%)	4(3.8)/66(63.5)/34(32.7)	3(6.8)/28(63.6)/13(29.5)	0.671
**GV (+/−), color, RC sign, *n* (%)**			
GV (+)/GV (−)	38(36.5)/66(63.5)	6(13.6)/38(86.4)	0.006
Cb/Cw	100(96.2)/4(3.8)	43(97.7)/1(2.3)	>0.999
RC0/RC1/RC2/RC3	16(15.4)/54(51.9)/29(27.9)/5(4.8)	6(13.6)/21(47.7)/13(29.5)/4(9.1)	0.777
LC (+/−)	98(94.2)/6(5.7)	39(88.6)/5(11.4)	0.304
HCC (+/−)	14(13.5)/90(86.5)	6(13.6)/38(86.4)	>0.999
**Blood biochemistry (median, range)**			
T‐bil level (mg/dL)	1.3 (0.4–4.5)	1.2 (0.6–4.5)	0.532
Alb level (g/dL)	3.4 (2.2–4.6)	3.4 (2.0–4.4)	0.645
AST level (U/L)	38 (15–241)	34 (19–112)	0.192
ALT level (U/L)	30 (8–170)	26 (10–62)	0.146
PLT count (10^3^/µL)	88 (23–291)	68 (22–224)	0.109
PT%	85 (44–115)	76 (52–115)	0.241
PT‐INR	1.1 (0.9–1.8)	1.2 (0.9–1.7)	0.113
Years of endoscopic experience (median, range)	13 (5–22)	8 (6–16)	< 0.001

Abbreviations: AIH, autoimmune hepatitis; Alb, albumin; ALT, alanine transaminase; AST, aspartate transaminase; Cb, blue varices; Cw, white varices; EPVO, extrahepatic portal vein obstruction; F, form (F1, small/straight; F2, enlarged/tortuous; F3, large/coil‐shaped); GV, gastric varices; HBV, hepatitis B virus; HCC, hepatocellular carcinoma; HCV, hepatitis C virus; IPH, idiopathic portal hypertension; LC, liver cirrhosis; Li, locus inferior; Lm, locus medialis; Ls, locus superior; mALBI, modified albumin‐bilirubin; MASH, metabolic dysfunction‐associated steatohepatitis; PBC, primary biliary cholangitis; PLT, platelet count; PT, prothrombin time; RC, red color sign (RC0, no redness observed; RC1, few localized lesions; RC2, between RC1 and RC3; RC3, multiple lesions observed around the entire circumference); T‐bil, total bilirubin.

**TABLE 2:** Comparison of post‐treatment adverse events in patients undergoing conventional endoscopic injection sclerotherapy (EIS) versus endoscopic injection sclerotherapy with ligation (EISL).

	All, 148 (%)	EIS group, 104 (%)	EISL group, 44 (%)	*p*‐Value
Chest heaviness/frontal chest pain	32 (21.6)	23 (22.1)	9 (20.4)	>0.999
Fever	25 (16.9)	23 (22.1)	2 (4.5)	0.008
Transient dysphagia	11 (7.4)	7 (6.7)	4 (9.1)	0.733
Hematuria	10 (6.8)	8 (7.7)	2 (4.5)	>0.999
Esophageal ulcer	10 (6.8)	9 (8.7)	1 (2.3)	0.451
Transient renal dysfunction	8 (5.4)	6 (5.8)	2 (4.5)	>0.999
Epigastric pain	7 (4.7)	6 (3.8)	1 (2.3)	>0.999
Nausea	6 (4.1)	4 (3.8)	2 (4.5)	0.631
Ascites	2 (1.4)	1 (1.0)	1 (2.3)	0.425
Renal dysfunction	2 (1.4)	2 (1.9)	0 (0)	>0.999
Esophageal submucosal hematoma	2 (1.4)	2 (1.9)	0 (0)	>0.999
Hiccup	1 (1.0)	0 (0)	1 (2.3)	0.243
Encephalopathy	1 (1.0)	1 (1.0)	0 (0)	>0.999
Skin rash	1 (1.0)	0 (0)	1 (2.3)	0.243
